# Memoing in qualitative research: two decades on

**DOI:** 10.1177/17449871251386323

**Published:** 2025-11-10

**Authors:** Melanie Birks, Ysanne Chapman, Karen Francis

**Affiliations:** Honorary Professor, College of Healthcare Sciences, James Cook University, QLD, Australia; Independent Scholar; Adjunct Professor, School of Nursing, Paramedicine and Healthcare Sciences, Charles Sturt University, NSW, Australia

**Keywords:** GenAI, Grounded theory, memoing, memos, nursing, qualitative research, technology

## Abstract

Memoing in research serves many purposes. In qualitative studies in particular, memoing supports research functions that facilitate decision-making, capture methodological activities, and propel a study forward. In 2008, we presented a framework for the use of memos in qualitative research, along with guidelines for developing and maintaining memoing as a practical strategy. In this paper, we revisit that original piece and consider the concept of memoing in support of research two decades on.

## Introduction

In a quote often attributed to Einstein, William Cameron (1957) once said ‘Not everything that can be counted counts’. For those of us whose careers are characterised by qualitative research, this is our driving belief. Unfortunately, it has long been the case that what cannot be counted often did not count for funding bodies, universities, and even scholarly journals. For much of the last century, qualitative researchers have struggled to demonstrate the legitimacy of their work. Glaser and Strauss’s (1967) gift of grounded theory gave us several tools that allow us to enhance and illustrate rigour in qualitative research. One such tool is memoing.

In 2008, *Memoing in qualitative research: Probing data and processes* was published in this journal. Now nearly two decades on, it is timely to revisit memoing and its use in qualitative research.

## Revisiting memoing

We produced the original paper during the first author’s PhD candidature. The second author had commented that the candidate ‘memos when she drops a pen’, given her commitment to memoing as a critical tool in the qualitative research process. In the years since, we have seen other students equally enamoured with memoing, yet there are many more who struggle to capitalise on its value as a procedural and analytical tool.

Memoing is powerful in qualitative research. It gives the researcher a sense of control over the research process. In the original paper, we discussed the role of memoing as a reflexive exercise that promoted engagement with the research. We proposed a mnemonic that captures the functions of memoing: ‘MEMO’ – Mapping research activities, Extracting meaning from the data, Maintaining momentum, and Opening communication. We also suggested some guidelines for the structure and content of memos. In the years following the publication of this work, what has changed?

The world has become a very different place in the two decades since the original paper was written, and this has implications for the way in which we conduct qualitative research. Some things have remained the same, however, including the responsibility of researchers to conduct research of significance, to do so ethically, and to disseminate it for impact. In this revisitation of our original work on memoing, we consider how our thinking has evolved in response to *methodological developments*, *technological developments*, and *pragmatethical developments* since its publication.

## Methodological developments

In our original paper, we discussed the concept of memoing in qualitative research generally; however, the examples used in that paper were drawn from a grounded theory study of nursing education. Memoing is most often associated with grounded theory, having been identified as an analytical technique by Glaser and Strauss in their seminal work (1967). Memoing no doubt existed prior to this early mention by these authors, if only as an extension of the anthropological field note. As is the case with many grounded theory methods, memoing has proved to be useful in other qualitative (and no doubt, quantitative) methodologies.

Although no figures are available that confirm the reliance on memoing by the various qualitative approaches, anecdotally we would suggest that the expansion of its use since the publication of our original paper in 2008 is notable. Our earlier work has been cited almost 3000 times. A rough desk-top analysis of the citation landscape of this original paper provides insight into methodologies that are employing memoing. Grounded theory and case study research were used in equal measure in the citing papers. About half this amount referred to phenomenology or ethnography, with less being about action research and narrative methodologies. The remaining papers that cited our earlier paper referred to discourse analysis, with a few papers mentioning historical research, or research methodologies in the general sense.

Of note, two-thirds of citing papers mentioned nursing, with the majority of those appearing to have used grounded theory methodology. This is not surprising given the popularity of grounded theory in nursing research. A little-known fact, even amongst grounded theorists, is that a nurse, Jeanne Quint Benoliel, was involved in the early work of Glaser and Strauss, with her support being deemed ‘invaluable’ by these authors in their original generation of the methodology (Glaser and Strauss, 1965: x). . Grounded theory is of course only one methodology that allows nurse researchers to investigate problems of social significance. Of interest is the value that memoing clearly holds for those conducting these studies.

## Technological developments

In the early days, Glaser (1978: 87) discussed the production of memos by hand or typewriter, although warned against the use of ‘machine’ sorting of memos. Two decades later, Glaser (1998: 186) continued to caution against the use of computers in grounded theory, although conceded that ‘computerization will likely catch up in the years to come, when accomplished grounded theory researchers turn their skills to generating software which helps not hinders the various stages of the grounded theory package’. This prophecy has arguably been fulfilled by the broader research community, with qualitative data analysis (QDA) software programmes such as *ATLAS.ti*, *MAXQDA* and *NVivo*, now in double digit versions.

There are likely those who may still prefer to produce memos using pen and paper; however, most qualitative researchers these days will do so electronically. For many decades, memos were produced using a word processing programme; however, QDA programs now have embedded memoing functionality. While documents produced externally can still be uploaded, QDA programmes enable researchers to produce and categorise memos that are either standalone or attached to data segments (Kalpokas and Radivojevic, 2022). Given that memos are themselves a type of data, this integration of memo functionality facilitates streamlined analysis of their content. These digital tools therefore make the process of developing and working with memos more efficient. One wonders, however, whether Glaser (1998: 185) would still consider that use of a QDA programme ‘numbs and stultifies these processes’.

In most recent times, we have seen the advancement of artificial intelligence (AI) and the large language models that have the potential to undertake a broad range of routine and creative tasks. AI now permeates every aspect of our lives (Shah, 2025), regardless of whether we seek to actively engage with it, or simply passively encounter it as we move through the world. We as nurses are familiar with the infiltration of technology generally, and AI specifically, in the healthcare context, but what about its use in research?

AI offers substantial assistance to researchers, from idea generation, through to data analysis, and report production. Standalone AI research assistant software such as *Elicit*, provides specific tools for researchers that reduce the laborious work associated with systematic reviews of the literature (Elicit, n.d.). General QDA programmes such as those referred to previously now incorporate AI into their functionality (Nguyen-Trung, 2025). We are all aware, of course, of the limitations of even the most advanced AI programmes, including issues with inaccuracies and hallucinations. It falls to us, therefore, to ensure we understand and work within these limitations.

Considering the strengths and weakness of AI as a research tool, what value does it have in respect of memoing? Can we outsource this critical methodological task to a computer programme? The short answer is no. The longer answer is that AI can value-add to the memoing process. Of all the memoing functions that we describe in our 2008 paper, maintaining momentum is arguably the most important. Memoing is a critical strategy in overcoming the inevitable ‘analysis paralysis’ (Clarke, 2005) experienced by researchers. AI has something to offer here.

AI is a massive neural network that works faster than the human brain and never forgets what it has learnt, and it had learnt *a lot* (Openmind Tech, 2025). Data analysis raises questions, which a researcher can attempt to answer in memos. Programs such as *ChatGPT* and *Copilot* are great to bounce ideas off. You can engage in a dialogue with AI that will open your eyes to alternative perspectives that you may not have otherwise considered, or that would have taken significantly more time to uncover through traditional means. Try dropping a memo or an extract of a memo into a program such as ChatGPT and ask it what it ‘thinks’. The response will be an analysis of your own ponderings, with some suggestions about how it aligns with, or can be extended by, existing knowledge. While this is useful in broadening your thinking, it can be particularly valuable in identifying theoretical frameworks in the early stages of a study for those methodologies that rely on these heuristics. Continuing the dialogue in this way throughout the research process can help to identify existing literature that can be used as data. In methodologies such as grounded theory, it may assist in identifying theoretical codes that add explanatory power to the final product.

## Pragmatethical developments

Pragmatethical developments are those that researchers face in ensuring their research remains ethical in the face of pragmatic realities. The term was coined by Microsoft Copilot, which is ironical, given that a number of these developments are related to the use of AI. Here, we use the term to capture practical and ethical challenges that exist in the contemporary research context. Many of these challenges stem from the increasing complexity of contemporary society, which has implications for the problems we study and the approach we take to investigating them. The most emergent issues, however, arise from the technological developments previously discussed.

Most researchers uphold the highest standard of ethics, yet are confronted by a rapidly changing landscape. Universities are increasingly driven by the bottom line. While indispensable, research is an expensive endeavour, and one that can be made more efficient through the use of digital tools. Many qualitative researchers are nervous about employing emerging technologies, yet failing to embrace them will see them left behind in contrast to their quantitative colleagues, whose objective stance in research has made the adoption of technology less pragmatethically challenging. There is therefore a tension between recognising the need to incorporate technologies such as AI into qualitative research work, and fearing that doing so would be inconsistent with the philosophical principles that underpin it.

Despite reservations held by some qualitative researchers, there are examples of the use of AI within this paradigm, particularly in respect of analysis (for recent examples see Jalali & Akhavan, 2024; Sinha et al., 2024; Nguyen-Trung, 2025; Pattyn, 2025; Zhang et al., 2025). Questions about the ability of AI to replace the interpretive role of the researcher persist, perhaps due to concerns amongst researchers that replacing the human element will undermine the true nature of qualitative work (Marshall and Naff, 2024). While we have discussed the potential for researchers to use AI to maintain momentum in their research, any use of AI for practical purposes can raise ethical issues.

Undertaking research from a relativist perspective, in an age where reality can be artificially generated, presents a new breed of challenges. Making the leap into the use of AI should therefore be done with attention to quality and authenticity. Rigour is demonstrated when decisions made and actions taken can be justified. Qualitative research can be deemed credible only when there is quality evident in both the processes and products of research. Memoing plays a role here in facilitating a record of quality.

While some researchers will choose to keep a log or diary of operational activities, we originally argued for the use of memos to map these crucial research activities (Birks et al., 2008). We propose that using memos provides a more comprehensive record than a simple audit trail. In the contemporary research environment, such attention to detail is essential. Memoing facilitates recording of not only decisions and actions themselves, but also the thought processes that lead to these decisions and actions. For example, a determination to use generative AI in a study (or not) is memo-fodder, as are the consequences of this choice. Through memoing, a researcher can resolve, or at least come to terms with, the internal (and external) conflict that arises from the methodological, technological, and pragmatethical developments they face in undertaking their work.

## Conclusion

Qualitative research has come a long way in the last century. We have fought battles on many fronts to legitimise the products of this tradition and for the most part, have won. In this fight, the ability to demonstrate rigour in this approach to research has been a crucial strategy, and memoing an important weapon. We made a case for the value of memoing in qualitative research in 2008. While the methodological, technological, pragmatic, and ethical developments of recent decades could weaken the position of qualitative research, given the centrality of the researcher in this paradigm, we reiterate the indispensability of memoing.

**Key points for policy, practice and/or research**

- Memoing serves a number of purposes in research.
- The role of memoing has become increasingly important in light of changes to the research landscape in recent decades.
- Memoing supports researchers in managing methogological, technological, pragmatic, and ethics challenges.
